# Navigating travel in Europe during the pandemic: from mobile apps, certificates and quarantine to traffic-light system

**DOI:** 10.1093/jtm/taac006

**Published:** 2022-02-03

**Authors:** Justine I Blanford, Nienke Beerlage-de Jong, Stephanie E Schouten, Alex W Friedrich, Vera Araújo-Soares

**Affiliations:** Faculty of Geoinformation Science and Earth Observation, University of Twente, Enschede, the Netherlands; Section of Health Technology and Services Research, Technical Medical Centre, University of Twente, Enschede, the Netherlands; Section of Health Technology and Services Research, Technical Medical Centre, University of Twente, Enschede, the Netherlands; Department of Medical Microbiology and Infection Prevention, University Medical Center Groningen, Groningen, the Netherlands; Section of Health Technology and Services Research, Technical Medical Centre, University of Twente, Enschede, the Netherlands

**Keywords:** Geographical spread, cross-border, vaccination, mobility, public health, adherence, health risk

## Abstract

**Background:**

Ever since 2020, travelling has become complex, and increasingly so as the COVID-19 pandemic continues. To reopen Europe safely, a consensus of travel measures has been agreed between countries to enable movement between countries with as few restrictions as possible. However, communication of these travel measures and requirements for entry are not always clear and easily available. The aim of this study was to assess the availability, accessibility and harmonization of current travel information available in Europe.

**Methods:**

We performed a systematic documental analysis of online publicly available information and synthesized travel entry requirements for all countries in the European Union and Schengen Area (*N* = 31). For each country we assessed entry requirements, actions after entry, how risk was assessed, and how accessible the information was.

**Results:**

We found varying measures implemented across Europe for entry and a range of exemptions and restrictions, some of which were consistent between countries. Information was not always easy to find taking on average 10 clicks to locate. Twenty-one countries required pre-travel forms to be completed. Forty apps were in use, 11 serving as digital certification checkers. All countries required some form of COVID-19 certification for entry with some exemptions (e.g. children). Nineteen percent (*n* = 6) of countries used the ECDC risk assessment system; 80% (*n* = 25) defined their own. Forty-eight percent (*n* = 15) of countries used a traffic-light system with 2–5 risk classifications.

**Conclusion:**

A comprehensive set of measures has been developed to enable continued safe travel in Europe. However further refinements and coordination is needed to align travel measures throughout the EU to minimize confusion and maximize adherence to requested measures. We recommend that, along with developing travel measures based on a common set of rules, a standard approach is taken to communicate what these measures are.

## Background

**Figure 1:**
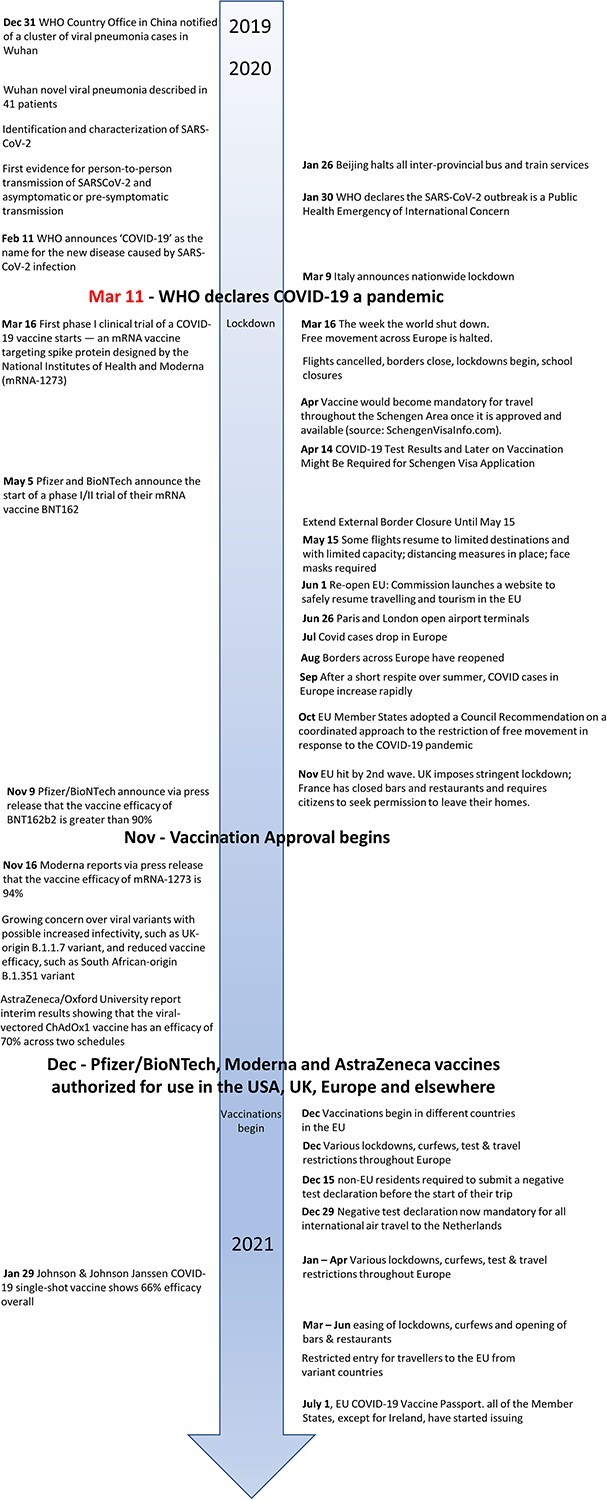
Timeline of events (sources: events^39–49^).

In January 2020 SARS-CoV-2, better known as COVID-19,^1^ spread globally astonishingly quickly. To halt the continued spread of the virus borders were shut, schools closed, travel stopped. The face-to-face (f2f) world came to a grinding halt and with that all cross-border movement ceased (see Figure 1 of a timeline of events). In March 2020 the entire world shutdown.^2^ All flights ceased and did not resume until May 2020 when borders slowly reopened and restrictions eased. In December 2020 vaccinations began along with test and travel restrictions (Figure 1). Since then, a set of measures have been developed, adopted and implemented to enable the safe reopening of Europe (Schengen Area and EU Member States) in a sustainable way.^3^^,^^4^

The measures consist of a set of common criteria useful for (i) assessing country-level risk, (ii) standardizing communication of risk (traffic-light approach), (iii) determining travel entry requirements, (iv) implementing travel measures and restrictions as well as (v) defining exemptions (summarized in Table 1)^4^^,^^5^ to minimize the hindrance of the movement of essential supplies and medical personnel.^6^ After all, *‘there is a stronger argument to keep trade [compared with passenger] links open to avoid food shortages. But countries must improve coordination and develop their capacity to fight infectious diseases*’.^6^

**Table 1:** Common approach to lifting restrictions and suggested travel measures to implement by each country

Overview	Definition	Criteria suggested by European Council
Assessing risk of a country	Criteria for assessing risk of a country	• The notification rate (the total number of newly notified COVID-19 cases per 100 000 population in the last 14 days at regional level). • The test positivity rate (the percentage of positive tests among all tests for COVID-19 infection carried out during the last week). • The testing rate (the number of tests for COVID-19 infection per 100 000 population carried out during the last week).
Communication of risk	Standard communication of risk	Five categories of risk were defined to communicate risk: • Green, if the notification rate is <25 and the test positivity rate is <4%; • Orange, if the notification rate is <50 but the test positivity rate is 4% or more, or, if the notification rate ranges from 25 to 150 but the test positivity rate is <4%; • Red, if the notification rate is 50 or more and the test positivity rate is 4% or more, or if the notification rate is >150; • Dark-red, if the notification rate is 500; • Grey, if not sufficient information is available or if the testing rate is 300 or less.
Travel requirements for entry	Entry requirements	Member States can decide what measures to apply on people travelling from risk areas to their territories. Options include: • Passenger locator form (PLF), in accordance with data protection requirements; • Require a RT-PCR or rapid antigen test (e.g. required: negative PCR test taken at the earliest 72 h prior to departure for person travelling for essential or non-essential reasons); • COVID-19 certification (vaccination, test or recovery).
Travel measures and restrictions	Travel with digital COVID-19 certificate	Member States have agreed that there will be no restrictions, such as quarantine or testing, on travellers coming from ‘green’ regions and those with EU Digital COVID Certificate. A COVID-19 certificate allows you to travel safely and freely within the European Union. Three distinct types of certificates have been defined to prove you are COVID-safe https://covidsafe.be/en/) and include: • A vaccination certificate proves that you have been fully vaccinated against the COVID-19 virus. • A test certificate proves that you have taken a negative COVID-19 test. • A recovery certificate shows that you have recovered from COVID-19 after a previous positive COVID-19 test.
	Travel restrictions: requirements	Member States can decide whether they introduce certain restrictions, such as quarantine or tests, on travellers coming from other areas. • Restrictions could be required for persons travelling from an area classified other than ‘green’ (e.g. undergo quarantine/self-quarantine; and/or take a test for COVID-19 infection before or after arrival.
	Travel restrictions and measures: self-isolation, quarantine and contact tracing	Quarantine and additional testing upon or after arrival should be imposed on travellers arriving from a third country where a variant of concern of the virus has been detected. Self-isolation, quarantine and contact tracing • For a period of up to 14 days; • Further COVID-19 testing as needed during the same period.
	Provision on children	Ensuring unity of travelling families and a standard validity period for tests.
Exemptions		• Essential functions—travellers with an essential function or need should not be required to undergo quarantine when arriving from an ‘orange’, ‘red’ or ‘grey’ area. While performing their duties. • Cross-border regions—people living in border regions should also be exempted from some of the travel restrictions. • If they frequently need to cross the border, for instance for family or work reasons, should not be required to undergo quarantine and the frequency of tests required should be proportionate. • If epidemiological situation on both sides of the border is similar, no testing requirement should be imposed.

Ultimately the goals have been to develop ways to protect the traveller and those around them.^7^^,^^8^ Mechanisms for reducing risk include minimizing the risk of acquiring, transmitting and spreading the virus by implementing precautions at all stages of travel (before, during and after) through detection and monitoring of infections.^8^^,^^9^ In the past year we have seen the development and implementation of many different travel measures. These have ranged from doing nothing to quarantining with some combination of testing and monitoring (see^8^^,^^9^). The effectiveness of these measures varies with when and how they are implemented (e.g.^10^). For example, pre-travel testing can reduce transmission risk by 10–29% and up to 44–72% with further testing on the day of travel.^9^ Testing before and after travel can provide an additional 37–75% reduction in transmission, as does quarantining at different time intervals (e.g. 96–100% reduction for a 14-day quarantine; 84–100% reduction for a 10-day quarantine; 64–95% reduction for a 7-day quarantine).^9^ Furthermore, a combined approach such as a 7-day quarantine with symptom monitoring and a single test conducted 5–6 days can result in a 97–100% reduction in transmission risk.^9^ Therefore, any measures implemented need to be evaluated for effectiveness in risk reductions, as demonstrated by.^9^ Adherence to travelling recommendations requires that communication on travel requirements is clear and easily understood, accepted and feasible to implement both by citizens as well as by the legal authorities responsible to supervise it.

The European Union and the Schengen Area is comprised of 31 countries with some 40 internal land borders and 30% of the EU population living in cross-border regions.^11^^,^^12^ Considering the complex geographic and border nature of Europe it is important to understand what information on how to travel between countries European citizens have access to. During the summer of 2021 travelling in Europe required individuals to wade through a minefield of information to ascertain whether one could travel and what was required to enter. The communication of these travel measures and requirements for entry were not always clear and easily available. Given the vast amount of information that was available on the topic of interest (e.g. entering another country, travelling through a country by different transport modes re-entering one’s home country), it was necessary to focus on a particular aspect of cross-border travel. Therefore, in this study we were interested in finding information on COVID-19 travel policies and requirements for entering each member country of the European Union or Schengen Area. Thus, the aim of this study was to assess the availability, accessibility and harmonization of current travel information available in Europe so that we could better understand what measures were implemented for entry, how much these varied between countries and what, if any, set of procedures could be used Europe-wide. In particular, we were interested in the availability of information, documentation requirements, measures required on arrival for entry and any exemptions for travel entry.

## Methods

A systematic documental analysis of online publicly available information was performed to synthesize travel entry requirements for all countries in Europe that are either a member of the European Union or part of the Schengen Area (*N* = 31 countries, Supplementary Table 3). First, the National Health Agency for each country was identified (see Supplementary Table 1 for the web addresses) and this information was used as the starting location from where information was collected. Since travel policies were continually changing information provided to the public, for this study, information was collected between 15 July and 2 August 2021.

Travel measures for each country were captured and evaluated in relation to the criteria defined in Table 1. For each country we assessed (i) availability and accessibility of travel measures: how easy was it to find the necessary information on travel measures; (ii) risk assessment: how was risk defined by each country and how was this used for defining and implementing travel measures; (iii) entry requirements: what was required to enter a country (before entry and at entry) for adults and children; and (iv) requirements after entry: what measures were in place after entry (e.g. additional testing and at what intervals, quarantine/self-isolation requirements) and what, if any exemptions were in place.

### Availability and accessibility of travel measures

Accessibility was measured in two ways: by capturing the total number of clicks and by capturing how many languages the information was made available in. Total number of clicks was captured by recording the total number of websites visited for each country to obtain travel information on entry requirements. Languages was captured for each country and broken down into two levels. Level one represents all the languages with information readily available and level two, additional languages available on request. Word clouds were created by analysing the languages using the open-source web-based Voyant Tools.^13^ For information that was not provided in English Google Translate was used to translate information into English. Some information for Estonia, Czech, Romania, Bulgaria, Croatia and Lichtenstein was translated.

### Risk assessment

**Figure 2:**
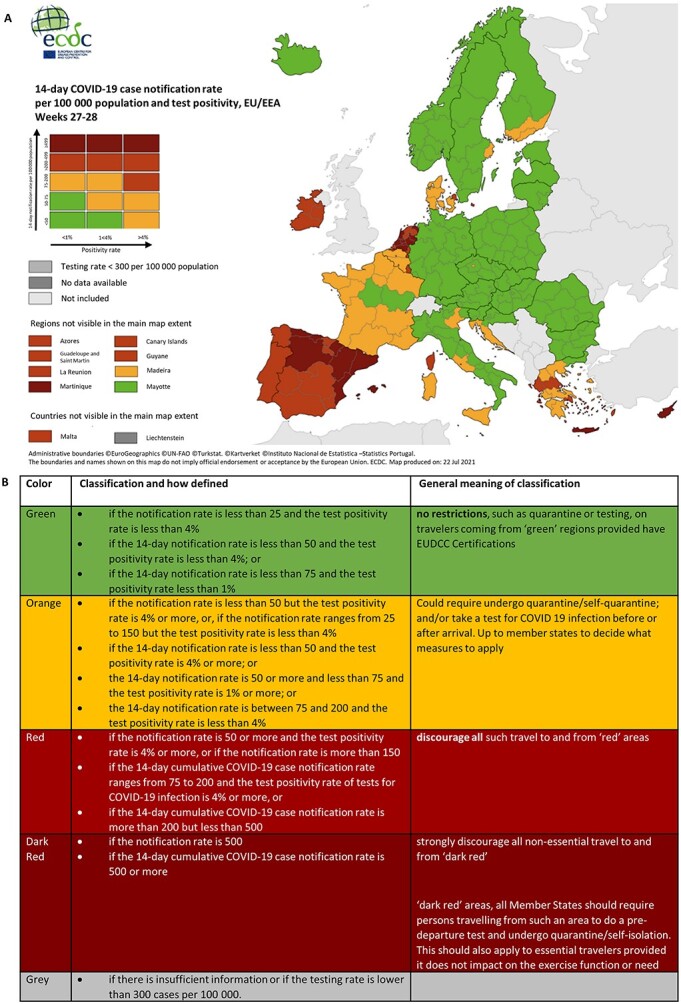
(A) Map illustrating the traffic-light approach used to capture weekly COVID-19 risk level in Europe and (B) the definition of each classification and suggested travel measure (source^4^^,^^50^: Map from 4 July and 11 July 2021).

Risk definitions were assessed as well as how risk classifications were used for communicating risk and defining travel measures. To complete this, key steps were followed: (i) evaluating how risk (e.g. notification rate, test positivity rate or testing rate, Table 1—criteria for assessing risk of a country) was defined for each country and how this matched the criteria defined by EU Commission (Table 1); (ii) comparing how risk was communicated for each country compared to the ECDC risk classification system (Table 1, Figure 2B). This was assessed by evaluating whether each country used the ECDC risk classifications (e.g. a traffic-light system; number of risk categories and how these compared to the classifications described in Figure 2B, Table 1); (iii) evaluating whether these risk classifications were used to define travel measures.

### Entry requirements

Information on what travellers were required to do to enter each country was compiled. Criteria for travel requirements (entry) as defined by the EU Commission (e.g. passenger locator form (PLF), pre-travel testing and COVID-19 certificates, Table 1) were gathered and based on this, a determination was made on whether countries used these criteria. Data were also collected on what additional requirements were required at entry (e.g. additional testing) and any exemptions.

### Actions after entry

Lastly, data were collected on what travel measures and restrictions were in place after country entry. To do so all available documents were examined to understand and code what travellers were required to do after entry. This included examining whether travellers were required to perform additional testing and at what time intervals; whether travellers were required to quarantine and if so for how long and under what conditions. Any exemptions were also recorded when in place.

## Results

### Availability and accessibility of travel measures

Data were collected from 310 different web pages, requiring on average 10 clicks (minimum = 5 and maximum = 18 clicks) to locate the necessary information. Information was available in 34 different main languages (Supplementary Figure 1A, Supplementary Table 2) with a further 76 languages available on request (Supplementary Figure 1B, Supplementary Table 2). All countries provided COVID-19 related travel information in their official language with 21 countries (68%) offering information in multiple languages. The most common additional language was English, which was offered by 21 countries (68%). Ten countries (32%) provided information only in a single language.

*Mobile apps*

A total of 40 COVID-19 mobile apps were in use to manage and monitor COVID-19 in Europe. Twenty-nine countries (94%) made use of one or more mobile apps to support safe cross-border travel. Most of the apps were designed to monitor risk through contact-tracing (57%). Twenty-two percent of the apps were used to store and retrieve COVID-19 certificates such as vaccination and testing. Less than 5% of the apps had multiple functions, serving as a symptom tracker or providing travel advice or information (Supplementary Table 3). Besides the national digital apps, two EU-wide systems were developed to (i) provide information on crossing borders, availability of means of transport, travel restrictions, public health and safety measures, and other practical information (i.e. Re-open EU) and (ii) store COVID-19 certificates (i.e. the EU digital COVID-19 certificate (EUDCC).

Two countries (6%) were either exploring the possibilities of development of an app (Romania) or had an app under development (e.g. Greece, contact-tracing app). Most (*n* = 22, 71%) countries only used a single app, some countries used two separate apps (*n* = 6, 19%) and two countries (6%, Germany and the Netherlands) had three different apps with complementary functionalities available (Supplementary Table 3).

### Risk assessment

Risk was based on a variety of information that included the epidemiological situation of a country (e.g. notification rate, incidence rate, positivity rate), prevalence of virus variants, vaccination status, geographic location (e.g. countries in the EU vs countries outside the EU), border traffic, security risk or the classifications defined by the ECDC (Table 1, Figure 2B). The criteria used for each country to assess risk was defined as a notification rate, incidence rate and/or positivity rate based on cumulative weekly or 14-days (Table 1). Six countries (20%) used the ECDC risk classifications (Figure 2A and B) for risk assessment. Twenty-five countries (80%) used their own risk assessment, some of which were based on the ECDC risk classifications. Fifteen countries (48%) used a traffic-light system with between 2 and 5 risk classifications (green, yellow/amber/orange, red, dark-red or grey). One country, Slovenia abolished the epidemiological risk assessment. Otherwise, the conditions of entry were the same for all countries.

The description of each risk classification (green, yellow/amber/orange, red, dark-red and grey) was not consistently defined between countries, nor were the travel restrictions associated with the risk classifications. About half (*n* = 16, 52%) of countries did not clearly define risk categories.

### Entry requirements

We found that a variety of information was required for entry. These included PLF, COVID-19 certificates and testing before travel (Table 2).

**Table 2:** Summary of travel measures implemented across Europe for each of the criteria suggested by the European Council defined in Table 1

Overview	Definition	Criteria implemented across the EU
Assessing risk of a country	Criteria for assessing risk of a country	Criteria used for assessing risk in a country included epidemiological risk (48%), variants of concern (26%), geographic location (16%), traffic (3%) or security risk (3%). Risk was evaluated for the 10 or 14 (=mode) days preceding travel.
Communication of risk	Standard communication of risk	Fifteen countries (48%) used a traffic-light system with between 2 and 5 categories to communicate risk. Six countries (19%) used the EUCDC risk assessment. Twenty-five countries (80%) used their own risk classification.
Travel requirements for entry	Entry requirements	**Passenger locator form (PLF)** Sixty-five percent of the countries required PLF’s to be completed within a specified time before arrival (24, 48 (=mode) or 72 h). **Exemptions for PLF** included occupational groups (6%), cross-border regions (10%), short stays (3%) or arriving from low epidemiological risk areas (3%). **Testing** All countries expressed the need for some form of testing. Eighty-three percent of the countries specified how long PCR tests were valid—(24, 48, 72(=mode) h) and 74% of the countries specified how long rapid test were valid (24, 48 (=mode), 72 h). **COVID certificates** All countries specified some form of COVID-19 certification (testing, vaccination or recovery) was required for entry. **Digital certificates and Apps** **EU Digital COVID Certificate (EUDCC):** All countries agreed to have the EU Digital COVID Certificate (vaccination, testing, recovery) to aid in minimizing restrictions. **Digital certificates & Apps:** Forty apps were developed for either contact tracing, symptom checker, certification checker, travel advice or information. **Non-EU Digital Certificates** were permitted by some countries and accepted by 16% (*n* = 5) of the countries. Thirteen percent (*n* = 4) of the countries required non-EU Digital Certificate documents to be translated in a specified language. Of these only one country accepted this if vaccinated. **Exceptions**—if entering from very high-risk area or an area with variants of concern (*n* = 1, 3%).
Travel measures and restrictions	Use of digital COVID-19 certificates	**COVID-19 certificates:** what is accepted and for how long—type of vaccine/testing accepted, effectiveness of vaccine, duration of certification (expiration of certificate): ***Vaccination Certificate***: ***Type***—EMA (32%), WHO (10%), emergency list WHO (3%) or specified (35%). ***Effectiveness of vaccination***—1 of 2 doses (21–90, 22–42, 22–84 days depending on vaccine) 2 of 2 doses (0, 1, 5, 7, 10, 14(=mode), 15 days after vaccination) 1 of 1 dose (0, 10, 14(=mode), 16, 21, 22, 28 days after vaccination) Recovered with 1 of 2 dose (7, 14(=mode), 21, 22 days after vaccination) ***Expiration of vaccination certificate**—*1 of 1 dose (180, 270, 365 (=mode) days) 2 of 2 doses (180, 270, 365 (=mode) days) ***Recovery certificate***: ***Effective of recovery certificate**—*valid from (10, 11 (=mode), 14, 20 days after positive test) ***Expiration of recovery certificate**—*valid for (180 (=mode), 270, 365 days) ***Test certificate***: ***Test type**—*EU approved, specified or none specified. ***Expiration of test certificate**—*PCR test valid for (24, 48, 72(=mode) hours) Rapid Test valid for (24, 48 (=mode), 72 hours) One country does not accept any rapid test certificates.
	Travel restrictions and measures: self-isolation, quarantine and contact tracing	Decisions for testing and quarantining were based on risk assessment, COVID-19 certification, geographic location of origin and traffic. **Testing**: several countries required testing on arrival (*n* = 17, 54%) or at set intervals (1,3,5,6,7 (=mode), 10, 14 days (*n* = 21, 68%)). **Risk assessment** Risk level of country arriving from (e.g. amber, red, dark-red); **Lacking COVID-19 certification**—not fully vaccinated; no COVID-19 certification; no testing prior to entry. **Geographic location**—non-contiguous territories; non-EU country; regardless of where travellers arriving from, all travellers must take a rapid test. Sweden specifies that all travellers arriving from outside Nordic countries must take a PCR test. **Quarantine/isolation** Seventy-one percent (*n* = 22) of the countries specified some form of quarantining requirement based on **risk assessment**—entering from high-risk areas; entering from areas with variant of concern; entering from grey risk areas; **lack of COVID-19 certification**—entering without being vaccinated or being fully vaccinated; or **geographic location**—non-contiguous country **Duration of quarantine:** length of time spent in quarantine ranged from 5 to14 days (mode = 10 days) depending on age, risk areas arriving from or testing before entry. Quarantine type ranged from self-isolation in suitable accommodation; mandatory hotel; government facilities to solitary confinement if test positive from arrival PCR test. **Shortening of quarantine** was possible (*n* = 15, 48% of the countries) based on a negative test at a specified time interval after arrival. Testing intervals ranged from 1 to 10 days (mode = 7 days). Exceptions to shortening quarantine were for those entering from areas with a variant of concern (*n* = 2, 6%). **Entry restrictions:** various restrictions were in place which ranged from no restrictions to those that only permitted entry for work, essential work or to visit family. Entry was prohibited for: tourists; people from variant areas; high-risk areas; without COVID-19 certification; unessential travel from very high-risk areas; unaccompanied minors; pregnant women without medical certificates.
	Provision on children	Exemptions of certificates for children ranged from 4 to 15 years of age (mode < 12 years).
Exemptions		Several different exemptions were in place for entry based on reason for travel, testing requirements, geographic location or COVID-19 certification. Certificate exemptions included: short stay (<12, 24, 48 (=mode), 72 hours); **Cross-border regions**—visiting areas within 30 km of the border; professional or educational requirements (e.g. international transport work, cross-border work, medical needs; essential business, education) **Exemptions on general entry:** Exemptions for all nationals or residents of a country; all nations or EU Members/Schengen Area Members from risk areas (green or amber or red or regardless of risk); all nations or residents or EU Members/Schengen Area Members COVID-19 certified; passing through or passing through without a stopover; agreement with EU member that share a border; members from green risk countries

*Passenger locator forms*

Twenty countries (65%) required travellers to fill in PLF. Sixteen of these countries (52%) explicitly specified for what modes of transportation such form was necessary (e.g. plane, car, public transportation or boat). Nine countries (29%) required a PLF for all modes of transportation; four countries (13%) required a PLF for travellers coming from high-risk level countries; two when travelling by car (6%); two when travelling by public transport (6%); three (10%) when travelling by boat; and five (16%) by plane. Eight of those who require a PLF (40%) also specified a timeframe in which the form must be completed. This ranged from 24 h to 72 h with a mode of 48 h.

Additional guidelines for PLF were provided by Estonia for travellers inbound by boat, car or public transport from areas of high-risk and Slovakia for cross-border workers in neighbouring countries. Exemptions for PLFs included travellers who were from areas of low epidemiological risk (*n* = 1, 3%), short-stays (*n* = 1, 3%) or specific occupational groups (*n* = 2, 6%).

*COVID-19 certificates*

All countries required COVID-19 certificates (test, vaccination or recovery). All 31 countries accepted the EUDCC as proof of vaccination, a negative COVID test or COVID recovery.

*Non-EU digital COVID-19 certificates*

Five countries (16%) also accepted digital health certificates from other countries; provided they were translated to pre-specified languages (except Malta) (*n* = 4, 13%). One country (3%) only accepted non-EU digital health certificates provided the traveller was vaccinated. Malta only accepted specific certificates (i.e. NHS pass, US CDC or certificates from the UAE, Serbia, Turkey, Guernsey, Jersey or Gibraltar).

Travellers in possession of valid EUDCC were still prohibited from entering Belgium when arriving from areas of very high-risk or from areas with variants of concern.

*Vaccination certificate*

Twenty-eight countries (90%) provided additional information about vaccines. This included *type—*what vaccines were approved (e.g. Pfizer, Moderna, etc.), *timing* of vaccinations—after how many days vaccinations were considered to become effective (e.g. number of days after dose) and *expiration* of certificate—how long vaccinations were valid for.

- *Type*. Twenty countries (65%) listed the vaccines that were accepted. These included vaccines approved (either full or emergency authorization approval) by the European Medical Agency (EMA) (*n* = 10, 32%), World Health Organization (WHO) (*n* = 3, 10%), WHO Emergency List (*n* = 1, 3%) or as specified by that country (*n* = 11, 35%).
- *Timing of vaccinations*. Twenty-six countries (84%) provided the number of days after which a vaccination was considered to be effective (50%–80% depending on vaccination type). For one of two doses this ranged from 21 to 90, 22 to 42, 22 to 84 days depending on vaccine); two of two doses ranged from 0, 1, 5, 7, 10, 14 and 15 days after vaccination (mode = 14 days); one of one dose ranged from 0, 10, 14, 16, 21, 22, 28 days after vaccination (mode = 14) and for recovered COVID-19 persons who have had one of two doses (i.e. considered fully immunized) ranged from 7, 14, 21, 22 days after vaccination (mode = 14).
- *Expiration of vaccination certificate*. Seven countries (23%) specified how long vaccination certificates were valid for. This ranged from 180, 270 to 365 days (mode = 365 days) for both one of one dose and two of two doses.

*Testing certificate*

Twenty-six countries (84%) provided additional information about tests. This included type—what tests were valid (e.g. PCR vs rapid antigen) and what tests were approved by the EU; expiration of test certificate—the timing of the test and how long the test results were valid for.

All countries expressed the need for some form of testing before arrival. Twenty-six countries (83%) specified how long PCR tests were valid. These ranged from 24, 48, 72 h (mode = 72 h). Twenty-three countries (*n* = 74%) specified how long rapid tests were valid. These ranged from 24, 48, 72 h (mode = 48 h). One country accepted only PCR tests (Ireland). Thirteen percent of countries specified what tests were valid.

*Recovery certificate*

Information on recovery certificates included timing and period of validity. Recovery certificates were valid from a set number of days after a positive test. Eleven countries (35%) included a day from which certificates were valid. This ranged from 10, 11, 14 and 20 days (mode = 11 days). Twenty-four countries (77%) defined the expiration of recovery certificates. These were valid for 180, 270 or 365 days (mode = 180 days).

*Children*

Twenty-eight countries (90%) included travel information for children. This included exemptions on testing and quarantining and the age at which testing was required for entry. The age ranged from 4 to 15 years of age (mode < 12 years). Three countries (10%) did not define exemptions for children.

*Entry restrictions*

Four countries (13%) listed a variety of restrictions. These ranged from no restrictions to those that were permitted entry for work, essential work or to visit family. In some cases, entry was prohibited for: tourists; people from new variant areas; people from high-risk areas; people without COVID-19 certification; unessential travel from high-risk areas; unaccompanied minors; pregnant women without medical certificates.

*Exemptions on general entry requirements*

Nine countries (29%) listed exemptions on general entry requirements in place. Exemptions varied and included: all nationals or residents of a country (*n* = 3, 10%); all nations or EU Members/Schengen Area Members from risk areas (green or amber or red or regardless of risk) (*n* = 2, 6%) provided they have a valid EUDCC and/or a negative PCR test (*n* = 1, 3%); whereas other members all nations or residents or EU Members/Schengen Area Members COVID-19 certified (*n* = 2, 6%); passing through or passing through without a stopover (*n* = 5, 16%); agreement with EU member that share a border (*n* = 1, 3%); members from green risk countries (*n* = 1, 3%).

### Actions after entry

Twenty-nine countries (94%) also included information about what travel measures were in place after entry. These included additional testing at set time intervals, self-isolation/quarantine requirements including the length of time and any exemptions for these measures.

All countries agreed that travellers from within the EU with a valid EUDCC would be allowed entry without being subject to further restrictions such as testing or quarantine/self-isolation. Switzerland and Liechtenstein will only accept this if travellers are not entering from areas with COVID-19 variants of concern.

The implementation and requirement for additional actions such as testing and quarantining after entry were determined through risk assessment and risk level of country arriving from or geographic location and/or COVID-19 certification or lack thereof. For example, travellers arriving from countries categorized with a risk level of amber, red or dark-red or from a specified geographic location (e.g. non-EU country or outside specified non-contiguous territories) may need to quarantine for a specified number of days and undergo testing at set intervals. The length of quarantine and ability to shorten time spent in quarantine will depend on the risk level of the country of origin. A similar approach is also applied to travellers who are lacking COVID-19 certification such as not being fully vaccinated, or not having been tested prior to entry.

Aside from entry restrictions for each country, travellers would further need to adhere to local measures that may be in place (e.g. within country travel, indoor and outdoor meetings, public or private gatherings and events, visits to touristic areas, museums and other public places).

*Testing*

Seventeen countries (54%) required testing on arrival. Twenty-one countries (68%) expressed the need for testing at specified time intervals. This ranged from 1, 3, 5, 6, 7, 10, 14 days (mode = 7). Decisions for testing and quarantining were based on risk assessment, COVID-19 certification, geographic location of origin and traffic. For some countries all travellers were required to take a rapid test regardless of where they were coming from, whereas Sweden required all travellers arriving from outside Nordic countries to take a PCR test.

*Quarantine/isolation*

Twenty-two countries (71%) specified some form of quarantining requirement based on risk assessment such as when entering from high-risk areas; entering from areas with a variant of concern; entering from grey risk areas where risk levels are unknown; lack of COVID-19 certification—entering without being (fully) vaccinated; or geographic location—entering from a non-contiguous country. The length of time spent in quarantine ranged from 5 to 14 days (mode = 10 days) depending on age, risk areas arriving from or testing before entry. Quarantine type ranged from self-isolation in a suitable accommodation, a mandatory hotel, government facilities or solitary confinement if one tested positive on entry from arrival PCR test. Shortening of quarantine was possible for 48% (*n* = 15) of countries based on a negative test at a specified time interval after arrival. Testing intervals ranged from 1 to 10 days (mode = 7 days). Exceptions to shortening quarantine were in place for those entering from areas with a variant of concern (*n* = 2, 6%).

*Exemptions*

Several different exemptions were in place for entry, based on reason for travel, testing requirements, geographic location or COVID-19 certification. Certificate exemptions included short stay visits where travellers entered the country for >12, 24, 48 or 72 h (mode = 48 h) (*n* = 5, 16%), or who remain within cross-border regions within 30 km of the border (*n* = 2, 6%); those who travel for professional or educational requirements (e.g. international transport work (*n* = 3, 10%), cross-border work (*n* = 2, 6%), medical needs (*n* = 1, 3%); essential business (*n* = 1, 3%), education (*n* = 2, 6%)).

## Discussion

Travelling today has become complex, requiring travellers to search for information on travel requirements and to assess what is required of them before and after entry. This is made more difficult when information is not communicated clearly. The aim of this study was to assess the availability, accessibility and harmonization of current travel information available in Europe.

Overall, results indicate that a wide variety of information was available for each country with some providing more information than others and in varying levels of detail. The information provided was not consistent between countries and in some cases required to be translated by the users themselves when they do not understand the country’s native language. The travel measures and restrictions for each country were not consistent nor communicated in a consistent manner, thus making it difficult to understand requirements for single or multi-staged journeys with and without family.

However, findings revealed that all countries used the criteria set out by the EU commission to establish their travel measures and restrictions and in many cases applied similar measures (e.g. acceptance of EUDCC, time limit and validity of PCR or rapid testing). Furthermore, all countries applied some form of combined approach (pre-travel testing and quarantine, with post-travel testing at set time intervals) to minimize risk and the importation of new virus cases. The approaches implemented are consistent with findings by^8^^,^^9^ who explored different approaches for safer travel.

Effects of cross-border measures and their role in the prevention of disease transmission or risk are still not well understood^14^ and are often conflicting. As captured in previous research,^14^ there are many reasons why travel measures on their own may not work as they may only delay the spread of the disease,^15^ be counterproductive,^6^^,^^16^ disruptive^17^ and avoid solving the problem (e.g. mitigation and suppression). We call for cross-European cooperation to allow for large scale evaluations of the effectiveness, but also of the understandability, acceptability and resulting adherence of and to cross-border travel measures. In doing so, attention should be paid to the measures themselves but also to the way they are communicated to the general public, as this will contribute to or hinder understandability, acceptability and resulting adherence.

Travel prevention measures can be effective in reducing imports. For example, COVID-19 infection rates in flight passengers varied between 3.6% and 6.3%.^18^ Travel prevention can hence contribute to limiting the spread of a disease (e.g. reduce spread by 40%^10^ and up to 92%^19^). However, it is the timing of the intervention^10^ or travel prevention measures paired with community efforts^20^ such as physical distancing^19^; quarantining^21–23^ and public health interventions^19^^,^^24^ that maybe more effective in reducing transmission and further outbreaks than preventing travel alone. Moreover, travel prevention measures contradict one of the fundamental rights of EU citizens: the right to free movement (across borders) as described in Article 45 of the Treaty on the Functioning of the European Union.^25^ These issues make it even more paramount to organize a EU-wide approach to cross-border travel during the COVID-19 (or in fact: any) pandemic.

It is clear that as more information becomes available, strategies for safe travel will need to be continually examined, adjusted and refined. This may include vaccine passports^26^ alongside quarantine and testing strategies (see^8^^,^^9^^,^^23^^,^^27^) to reduce imports^18^ and transmission during travel^23^ and minimize infections during flights. Similarly, as more information becomes available on the viability and longevity of vaccines adjustments in expiration of certificates (e.g. 365 days) and when one becomes fully vaccinated (e.g. after 7 or 14 days for the second of two doses and after 21 days for single doses^28^^,^^29^) can and should be made into a standardized Europe-wide policy to allow for easier cross-border travelling. After all the same vaccines are being used that are approved at multiple levels—the WHO^30^ and the European Medicines Association.^31^

What was less clear was how each country assessed risk and how these assessments were used for implementing and determining travel restrictions and measures. Due to this variation, it was not clear what this meant for travelling. A traffic-light system with five classifications was implemented by the ECDC (Figure 2) to simplify communication of risk. However, this was not consistently applied by all countries, with many countries defining and using their own classification scheme. This inconsistency can result in confusion, making it difficult for travellers to determine what measures and restrictions are in place but also to adhere to said requirements.

On the use and integration of technologies such as apps for monitoring symptoms, contact-tracing, providing information and for the digitalization of certificates, it seems clear that further refinements are needed to ensure continued integrity, data protection and adherence (e.g.^32^^,^^33^) as well as the ability to integrate foreign COVID-19 certificates and different COVID-19 vaccination types so as to allow for continued safe travel, minimize confusion and ensure equality and prevent discrimination. After all, as Dr Tedros Adhanom Ghebreyesus recently stated: ‘*some countries are refusing entry to people who have been fully vaccinated with a vaccine that has WHO Emergency Use Listing, but which has not been approved by their own national regulators. This is creating more chaos, confusion and discrimination, with some countries even refusing to use certain vaccines because of concern their citizens will be denied entry to other countries. WHO Emergency Use Listing follows a rigorous process based on internationally recognized standards. All vaccines that have received WHO emergency use listing are safe and effective in preventing severe disease and death, including against the delta variant*.’^34^

Although Europe encourages the free movement and flow of goods and people, closed borders and travel restrictions during the pandemic have restricted flows (see^17^ for details and examples) resulting in economic and social impacts (see^14^). Exemptions were specified (e.g. continued access for work, medical treatment or education) some of which extended to a 30 km zone from the border. With some 40 internal borders and 30% of the EU population living within cross-border regions,^11^^,^^12^ greater clarity and consideration of locally established cross-border cooperation is required within these regions when devising travel measures.

The European Commission has been efficient in developing comprehensive guidelines for a dynamically changing and evolving situation (Table 1). However, it is not clear who is involved and how these criteria are established or how information flows. Thus, we argue that having a supra national organization with the mandate to supervise and coordinate communication between countries, especially those sharing borders, would facilitate the communication amongst these and would allow for the issuing of streamlined regulations, easier to understand and adhere to by the population as well as easier to implement and audit for governmental and healthcare institutions. Greater levels of adherence to these measures would go a long way to defend the health of the populations and to deal with the spread of COVID-19 virus and its new variants more effectively, or any potential future pandemic. The proposal is that clear procedures need to be drawn to increase communication between different country agencies if we are to have more efficient responses to high-impact cross-border events such as pandemics.

The results on this paper strengthen the recommendations by the Pan European Commission on Health and Sustainable Development. This commission was convened by WHO Europe as a response to the COVID-19 pandemic, its 19 members are leaders in the political, economic and scientific fields. Their aim was to propose better ways for governments to respond to pandemics, considering that the European region is both remarkably diverse as well as very interconnected. On their report^35^ they call for a Pan European Health Threats Council to maintain political commitment and cooperation and for a Network for Disease Control to respond to emerging threats. They also call for the Creation of a Global Health Board (under the G20), a Pandemic Treaty for joint decision making as well as a Global Pandemic Vaccine policy.

Considering what has happened during this pandemic and analysing the data that was collected and presented here, it is true that no country can do it alone.^36^ It is time to build organizational infrastructures that allow the EU and its Schengen area to be nimbler and more flexible when dealing with crises like this COVID-19 pandemic. The aim will be to reduce human, social and financial costs and to facilitate individuals, families and communities’ adherence to measures implemented in an area that has been, in effect, borderless for several decades.

**Table 3:** Recommendations for standardization of travel measures

Travel measure	Criteria	Details	Suggested standardization
Entry requirements	Pre-entry travel form	Standard (digital) form for ALL modes of travel to be completed within 48 h of travel	Standardized forms for all EU Member countries and the Schengen Area.
			A centralized standardized multi-lingual EU app should be used.
	COVID-19 certificate	Standardized proof of negative test, recovery or full vaccination	EUDCC and approved digital certificates from countries outside the EU should be accepted everywhere. Non-digital forms should also be accepted.
			For non-EUDCC and non-digital forms translations should be available into the national language of destination or English.
			It should be possible to upload non-EUDCC and non-digital forms to EUDCC.
Health certificates	Test	PCR or rapid antigen tests approved by EMA and WHO	Test certificate should be required for all unvaccinated children >11 years (or minimum age that children are able to be vaccinated in home country).
			Pre-testing should be used based on scientific findings, mode of transportation and vaccination status of individual to minimize risk. All unvaccinated persons travelling should be tested regardless of mode of transportation. Vaccinated persons should be tested based on mode of transportation and risk classification of place of origin.
	Vaccinations	Vaccinations approved by EMA and WHO	Vaccinations should be valid based on scientific findings for each vaccine type and standardized across all countries.
	Recovery	Recovery from COVID-19	Standardize validity of recovery certificates
Risk assessment	Traffic-light system risk classification	Five risk classifications	A standard Europe-wide approach is needed for defining risk and what each of the risk classifications mean for travel and the implementation of travel measures.
			Risk classifications should include epidemiological risk level (see Table 1, Figure 2) and variant type.
			Risk assessment should be based on a set number of days prior to entry and should represent the transmission window of the virus (e.g. 10–14 days). This should be reassessed with different variants and standardized.
			Should I stay, or should I go? Clearly define risk classifications and what this means for travel measures as defined by ECDC. • Green—travel permitted. No restrictions on entry provided that the traveller has valid health certificates. • Amber/yellow/orange—Travel permitted provided that the traveller has valid health certificates. Some restrictions on entry may apply. • Red—travel permitted provided that the traveller has valid health certificates. Restrictions apply on entry. • Dark-red—non-essential travel restricted; restrictions apply. Entry is permitted with severe restrictions (combined approach—pre-testing; symptom-monitoring-quarantine-post-testing). Entry only for nationals and permanent residents, essential workers, work requirements, education requirements and medical requirements; Member of EU Country and Schengen Area but with restrictions on entry. • Grey—unclear risk due to incomplete data. Travel restrictions apply until individual assessment completed.
Exemptions	Essential workers and business		Continued exemptions for essential work and business cross-border travel to ensure the continued transport of essential goods and services.
	Border region		With close to 40 internal cross-border regions in Europe^11^,^12^; greater cooperation with established cross-border cooperations is necessary to ensure travel measures comply with regulations and agreements already in place. Exemptions from testing and quarantining for travel within 30 km of borders and within cross-border regions should be in place for all travel for work, essential transportation of goods and services, education or medical requirements.
	Transit		Continued exemptions for travel transit such as layover/stopover, driving through without stopping.
Additional	Information	Management and availability of travel measures and information	Travel measures: travel measures for Europe should be standardized, centralized, up-to-date and easily available and accessible (e.g. EU-ReOpen COVID-19 App,^51^ IATA^52^).
			Travel entry information for each country. Information for each country should be available in as few clicks as possible. Preferably also integrated in a centralized, single digital European COVID-19 platform.
			Contact information. Essential contact information for National Health Agencies or COVID-19 should be easily available so travellers know what to do if they start to develop symptoms.
			Travel restrictions: Clearly presented information on travel restrictions in place should be easily available for each country.
			Digitalization of information and certificates. Digitalization should be prioritized since it contributes to smooth cross-border transitions. However, this should not exclude those without digitalized documents.
			Information should be made available in several languages.

## Limitations

As with any scientific study, the current study had some limitations. This study provided a snapshot of the travel measures in place during the summer of 2021. Due to the evolving situation of the virus, changing infection rates, vaccination campaigns and knowledge thereof changed at a very rapid pace throughout 2021. To make sure that our data could be compared between countries, all data were collected within as narrow a time span (between 15 July and 2 August 2021) as possible. Since measures were constantly changing and being updated, this does mean that if this study were to be repeated using the current information, the results will likely differ. However, given the confusion that still exists when seeking and evaluating travel measures, now that the winter travel season is approaching with holidays and winter sports season, we are convinced that our key findings and recommendations are still very relevant and applicable.

A second limitation is a direct consequence of data both in terms of complexity and overwhelming amount of information. Throughout this pandemic, the general public has continually been confronted with enormous amounts of information about COVID-19. It is with good reason that this pandemic has also been referred to as an ‘*infodemic’.*^37^ This study was also affected by an overwhelming amount of information as has been recorded by the total number of unique websites (*N* = 310) from where information was retrieved for 31 countries. Much of the data were unstructured, that is, the data were heterogeneous and not available in a standardized format with tagged keywords. Information was initially found through National Health Agency websites and then through snowballing via links found on these websites. To the best of our knowledge we obtained all relevant information, but it is likely some information may have been missed. In addition, information may have been missed due to lack of clarity of information or the need for information to be translated (see Supplementary Table 2 for languages in which information was available). Regardless, we feel that the information presented here captures the travel measures available at the time and if information is missing then it further highlights the need for clarity and making this information available in structured, standardized and accessible formats. After all, key to the success of any public (health) information is that it is easily accessible and understood by all.

## Conclusion

In conclusion, our findings suggest that many countries have adopted a common approach with some variations. We suggest that the commonalities are used to create a standardized set of criteria that countries can use to communicate travel measures and restrictions that ensure safe travel while harmonized, as summarized in Table 3. This set of criteria needs to be evidence based and updated as new scientific knowledge becomes available. Variations in measures should be further examined and evaluated to determine if these are unique to a single geographic location or country, or whether they are also applicable to all countries and used to develop exemptions. Future work should include engagement with end users to guide the building of acceptable and feasible communication strategies as well as account for cultural country differences.^38^ Some of the organizational infrastructures proposed by the Pan European Commission on Health and Sustainable Development could make use of these recommendations.

## Authors’ contributions

JIB, NBJ, VAS, AF conceptualized and designed the study; SES performed the data collection; JIB, NBJ, VAS and SES analysed and interpreted the data. JIB, NBJ, VAS coordinated, drafted the initial manuscript; searched the literature, created the figures. All authors contributed to the writing of the paper.

## Conflict of interest

None declared.

## Supplementary Material

CrossBorderTravelEU_SuppInformation_2021_12_9final_taac006
