# Supplementary material for: Navigating travel in Europe during the pandemic: from mobile apps, certificates and quarantine to traffic-light system
Source: J Travel Med. 2022 Feb 3;29(3):taac006. doi: 10.1093/jtm/taac006 (PMC9155998; doi:10.1093/jtm/taac006)
Supplement: CrossBorderTravelEU_SuppInformation_2021_12_9final_taac006 [file crossbordertraveleu_suppinformation_2021_12_9final_taac006.docx]

**Supplementary Information**

**Supplementary Table 1:** Overview of Schengen & EU Countries and key National Health Authorities.

| **Country** | **National Public Health Name** | **National Public Health URL** |
| --- | --- | --- |
| Austria | Bundesministerium für Soziales, Gesundheit, Pflege und Konsumentenschutz | [https://www.sozialministerium.at](https://www.sozialministerium.at/) |
| Belgium | Service Public Fédéral Santé Publique; Federale Overheidsdienst Volksgezondheid; Föderaler Öffentlicher Dienst; Federal Public Service Health | <https://www.health.belgium.be/en> |
| Bulgaria | Ministry of Health | <https://www.mh.government.bg/en/> |
| Croatia | Institute of Public Health | <https://www.hzjz.hr/priopcenja-mediji/koronavirus-najnoviji-podatci/> |
| Cyprus | Ministry of Health | <https://www.moh.gov.cy/moh/moh.nsf/All/0D5A0919CACA4BF8C225851B003E098C> |
| Czech Republic | Ministry of Health | <http://www.mzcr.cz/Info.aspx?aspxerrorpath=/DokumentDetail.aspx> |
| Denmark | Sundhedsstyrelsen | <https://www.sst.dk/en/english> |
| Estonia | Terviseamet | <https://www.terviseamet.ee/en> |
| Finland | Finnish Institute for Health and Welfareh | <https://thl.fi/en/web/thlfi-en> |
| France | Ministère des Solidarités et de la SantéSanté Publique France | <https://solidarites-sante.gouv.fr/> |
| Germany | Bundesinstitut für Risikobewertung (BfR) Robert-Koch-Institut (RKI)Bundeszentrale für gesundheitliche Aufklärung (BzgA)Bundesgesundheitsministeriumht | <https://www.rki.de/> |
| Greece | National Public Health Institute of Greece | <https://eody.gov.gr/> |
| Hungary | Nemzeti Népegészségügyi Központ | <https://www.nnk.gov.hu/> |
| Iceland | Ministry of Health | <https://www.government.is/ministries/ministry-of-health/> |
| Ireland | Health Service Executive (HSE) | <https://www2.hse.ie/> |
| Italy | Istituto Superiore di Sanità https://www.iss.it | <http://www.salute.gov.it/> |
| Latvia | Centre for the Prevention and Control of Disease | <https://arkartassituacija.gov.lv/> |
| Liechtenstein | Ministry of Society and Culture | <https://www.regierung.li/coronavirus> |
| Lithuania | National Public Health Center under Ministry of Healt | <https://nvsc.lrv.lt/> |
| Luxembourg | Ministry of Health | [https://msan.gouvernement.lu](https://msan.gouvernement.lu/) |
| Malta | Government of Malta | [https://deputyprimeminister.gov.mt](https://deputyprimeminister.gov.mt/) |
| Netherlands | RIVM | <https://www.rivm.nl/> |
| Norway | Helsedirektoratet /The Norwegian Directorate of Health | <https://helsenorge.no/> |
| Poland | Ministerstwo Zdrowia (Ministry of Health);  Państwowa Inspekcja Sanitarna (State Sanitary Inspection) | [https://www.gov.pl](https://www.gov.pl/) |
| Portugal | Directorate General of Health, Ministry of Health | <https://www.dgs.pt/> |
| Romania | Centrul National de Supraveghere si Control al Bolilor Transmisibile; Comitetul Național pentru Situații de Urgența | [http://www.cnscbt.ro/; http:/www.dsu.mai.gov.ro/](http://www.cnscbt.ro/;%20http:/www.dsu.mai.gov.ro/)’  <http://www.ms.ro/> |
| Slovakia | Úrad verejného zdravotníctva Slovenskej republik  Public Health Authority of the Slovak Republic | <http://www.uvzsr.sk/> |
| Slovenia | Government of Slovenia | <https://www.gov.si/> |
| Spain | Government of Spain | <https://www.mscbs.gob.es/> |
| Sweden | The Public Health Authority of Sweden | <https://www.folkhalsomyndigheten.se/> |
| Switzerland | Federal Office of Public Health FOPH | <https://www.bag.admin.ch/> |

**Supplementary Table 2:** Overview of countries in the Schengen Area and/or a member of the European Union; countries with which each country borders, number of countries with which each country borders and languages used to provide travel information.

| **Country** | **Schengen country** | **Member of EU** | **Border Countries** | **Border Country Number** | **Main Languages** | **Additional Languages on request** |
| --- | --- | --- | --- | --- | --- | --- |
| **Austria** | yes | yes | Germany, Switzerland, Slovenia, Hungary, Slovakia, Czech Republic, Liechtenstein | 7 | German; English | Turkish; Croatian; Serbian; Bosnian; Romanian |
| **Belgium** | yes | yes | Germany, Netherlands, Luxembourg, France | 4 | Dutch; French; German; English |  |
| **Bulgaria** |  | yes | Romania, Turkey, Greece, North Macedonia, Serbia | 5 | English; Bulgarian |  |
| **Croatia** |  | yes | Bosnia and Herzegovina, Serbia, Hungary, Slovenia | 4 | Croatian | English, German, Italian, Czech, French, Japanese, Hungarian, Dutch, Polish, Russian, Slovakian, Slovenian, Spanish, Swedish |
| **Cyprus** |  | yes |  | 0 | English; Greek | French, Arabic, Somali, Persian, Georgian, Russian, Sinhala, Tamil, Vietnamese, Kurdish, Filipino, Kurmanji, Urdu, Bengali, Turkish, Hindi, Sorani, Chinese, Romanian, Bulgarian |
| **Czech Republic** | yes | yes | Germany, Poland, Slovakia, Austria | 4 | Czech |  |
| **Denmark** | yes | yes | Germany, Sweden | 2 | Danish; English | Arabic, Bosnian, Serbo, Croatian, English, Farsi, French, Kurmanji, Polish, Romanian, Somali, Tigrinya, Turkish and Urdu |
| **Estonia** | yes | yes | Latvia, Russia, Finland | 3 | Estonian; English; Russian |  |
| **Finland** | yes | yes | Russia, Estonia, Sweden | 3 | Finnish; Swedish; English |  |
| **France** | yes | yes | Switzerland, Italy, Luxembourg, Belgium, Spain | 5 | French |  |
| **Germany** | yes | yes | Netherlands, Czech Republic, Austria, Switzerland, France, Belgium, Luxembourg, Denmark, Poland | 9 | English; German |  |
| **Greece** | yes | yes | Albania, North Macedonia, Bulgaria, Turkey | 5 | Greek; English |  |
| **Hungary** | yes | yes | Austria, Slovakia, Romania, Serbia, Bosnia and Herzegovina, Croatia, Slovenia, Ukraine | 8 | Hungarian |  |
| **Iceland** | yes |  |  | 0 | Icelandic; English | Arabic, German, Spanish, Farsi, French, Kurdish, Lithuanian, Polish, Talelenska |
| **Ireland** |  | yes | UK | 1 | English; Gaelic | Albanian, Amharic, Arabic, Bengali, Bulgarian, Chinese (Mandarin), Croatian, Czech, Farsi, Filipino, French, Georgian, German, Greek, Hindi, Hungarian, Italian, Kurdish Sorani, Latvian, Lingala, Lithuanian, Macedonian, Moldovan, Pashto, Polish, Portuguese, Romanian, Russian, Slovak, Somali, Spanish, Swahili, Tamil, Tigrinya, Ukrainian, Urdu, Yoruba, Zulu and accessible formats (easy read, deaf and hard hearing, Irish sign language) |
| **Italy** | yes | yes | France, Switzerland, Austria, Slovenia | 4 | Italian | English |
| **Latvia** | yes | yes | Estonia, Lithuania, Belarus, Russia | 4 | Latvian | Russian; English |
| **Liechtenstein** | yes |  | Switzerland, Austria | 2 | German; English |  |
| **Lithuania** | yes | yes | Latvia, Belarus, Poland | 3 | Lithuanian; English | Russian; Polish |
| **Luxembourg** | yes | yes | Belgium, Germany, France | 3 | French; German; English; Luxembourg |  |
| **Malta** | yes | yes |  | 0 | English |  |
| **Netherlands** | yes | yes | Germany, Belgium | 2 | Dutch; English |  |
| **Norway** | yes |  | Sweden, Finland, Russia | 3 | English; Norweign; Bokmål; Sámi | Amharic, Arabic, Estonian, Farsi; French; German; Greek; Lithuanian; Pashto; Polish; Romanian; Russian; Somali; Sorani; Spanish; Tigrinya, Turkish; Urdu |
| **Poland** | yes | yes | Germany, Czech Republic, Slovakia, Ukraine, Belarus, Lithuania | 6 | Polish | Russian; Ukranian; English |
| **Portugal** | yes | yes | Spain | 1 | Portuguese | English, Arabic, Chinese, Dutch, French, German, Italian, Japanese, Russian, Spanish |
| **Romania** |  | yes | Moldova, Ukraine, Hungary, Serbia, Bulgaria | 5 | Romanian |  |
| **Slovakia** | yes | yes | Poland, Ukraine, Hungary, Austria, Czech Republic | 5 | Slovakian; English |  |
| **Slovenia** | yes | yes | Austria, Hungary, Croatia, Italy | 4 | English; Slovenian; Italian; Magyar |  |
| **Spain** | yes | yes | Portugal, France | 2 | Castellano, Català, Euskara, Galego, Valencià, English, French |  |
| **Sweden** | yes | yes | Norway, Finland, Denmark | 3 | Swedish; English | Amharic; Arabic; Chinese; Dari; Finnish; French; German; Kurmanji; Pashto; Persian; Polish; Russian; Somali; Sorani; Spanish; Thai; Tigrinyan |
| **Switzerland** | yes |  | Germany, Austria, Liechtenstein, Italy, France | 5 | German; French; Italian; English |  |

**Supplementary Table 3:** Overview by country of COVID-19 mobile apps and their application (Sources include URL in table and [1].

| **Country** | **Mobile App** | **Contact Tracing App** | **Testing and vaccination certification checker** | **Other information** | **URL** |
| --- | --- | --- | --- | --- | --- |
| Austria | Stopp Corona app | Y |  |  | <https://www.austria.info/en/service-and-facts/coronavirus-information/app> |
| Belgium | CovidSafeBE app |  | Y |  | <https://covidsafe.be/en/> |
|  | Coronalert | Y |  |  | <https://coronalert.be/en/> |
| Bulgaria | ViruSafe | Y |  | Symptom tracker | <https://virusafe.info/> |
| Croatia | [Stop COVID-19](https://www.koronavirus.hr/stop-covid-19-723/723) | y |  |  | [Stop COVID-19 (koronavirus.hr)](https://www.koronavirus.hr/stop-covid-19-723/723) |
|  | CovidGO app |  | Y |  | <https://vlada.gov.hr/news/bozinovic-covidgo-free-mobile-phone-app-available-as-of-wednesday/32383> |
| Cyprus | [CovTracer-EN](https://urldefense.com/v3/__https:/covtracer.dmrid.gov.cy/__;!!DOxrgLBm!RbcdDHg75HqIz1NG_dRSoqTuG__94m37YgiZCKW7UDm4KOIbaGL0ss1_xykIOu1KQSfuNqXeJ80CSQQ$) | y |  |  | [COVTRACER - Republic of Cyprus \| Republic of Cyprus Presidency (dmrid.gov.cy)](https://covtracer.dmrid.gov.cy/dmrid/covtracer/covtracer.nsf/home_el/home_el?opendocument) |
| Czech Republic | ČTečka |  | Y |  | <https://praguemorning.cz/new-application-for-checking-vaccination-certificates-and-covid-19-tests-to-appear-in-the-czech-republic/> |
|  | eRouška | Y |  |  | <https://erouska.cz/en> |
| Denmark | Smittestop app | Y |  |  | <https://smittestop.dk/about-the-app/> |
|  | Coronapas |  | Y |  | <https://en.coronasmitte.dk/corona-passport> |
| Estonia | HOIA | Y |  |  | <https://hoia.me/en/> |
| Finland | Koronavilkku | Y |  |  | <https://koronavilkku.fi/en/> |
| France | TousAntiCovid | Y | Y |  | <https://www.gouvernement.fr/info-coronavirus/tousanticovid> |
| Germany | Corona-Warn-App | Y |  | Travel advice | <https://www.rki.de/EN/Content/infections/epidemiology/outbreaks/COVID-19/CWA/CWA.html;jsessionid=874D6A8025BFC3486ED29967D04A98C8.internet052> |
|  | The CovPass app |  | Y |  | <https://digitaler-impfnachweis-app.de/> |
|  | Covid Free GR app |  | Y |  | <https://digitaler-impfnachweis-app.de/>    <https://play.google.com/store/apps/details?id=gr.gov.dcc.mini&hl=en&gl=US> |
| Greece | Contact tracing app under development. |  |  |  | <https://ec.europa.eu/info/live-work-travel-eu/coronavirus-response/travel-during-coronavirus-pandemic/mobile-contact-tracing-apps-eu-member-states_en> |
| Hungary | Virus Radar app | Y |  |  | <https://virusradar.hu/> |
| Iceland | The Rakning C-19 app | Y |  |  | <https://www.covid.is/app/en> |
| Ireland | Digital COVID Certificate Checker |  | Y |  | [gov.ie - How to use the Digital COVID Certificate Checker (www.gov.ie)](https://www.gov.ie/en/publication/b1dc9-how-to-use-the-digital-covid-certificate-checker/?referrer=http://www.gov.ie/dccchecker/) |
|  | COVID Tracker app | Y |  |  | [gov.ie - How to use the Digital COVID Certificate Checker (www.gov.ie)](https://www.gov.ie/en/publication/b1dc9-how-to-use-the-digital-covid-certificate-checker/) |
| Italy | Immuni | Y | Y |  | <https://www.immuni.italia.it/> |
| Latvia | Apturi Covid App | y |  |  | <https://covid19.gov.lv/en/covid-19/safety-measures/apturi-covid-app> |
| Liechtenstein | SwissCovid app | Y |  |  | <https://www.llv.li/inhalt/118866/amtsstellen/swisscovid-app> |
| Lithuania | CORONA-STOP-LT APP | y |  |  | <https://nvsc.lrv.lt/en/information-on-covid-19/corona-stop-lt-app> |
| Luxembourg | CovidCheck.lu | The deployment of a contact tracing app is not foreseen | Y |  | <https://covid19.public.lu/en/covidcheck/app.html> |
| Malta | COVID Alert | Y |  |  | <https://covidalert.gov.mt> |
| Netherlands | CoronaCheck app |  |  |  | <https://www.government.nl/topics/coronavirus-covid-19/covid-certificate/covid-certificate-for-travel-or-events> |
|  | Coronamelder app | Y |  |  | <https://www.coronamelder.nl/nl/> |
|  | De Reis app van Buitenlandse Zaken en Douane |  |  | Travel Advice | <https://www.nederlandwereldwijd.nl/documenten/vragen-en-antwoorden/reis-app-buitenlandse-zaken> |
| Norway | The Smittestopp app | Y |  |  | <https://www.helsenorge.no/en/smittestopp/> |
| Poland | ProteGO Safe | Y |  |  | <https://www.gov.pl/web/protegosafe> |
| Portugal | Stayaway COVID app | Y |  |  | <https://stayawaycovid.pt/landing-page/> |
| Romania |  | Romania is exploring the development of a contact tracing app. |  |  | [1] |
| Slovakia | Covid19 ZostanZdravy | Y |  |  | <https://www.old.korona.gov.sk/en/COVID19-ZostanZdravy.php> |
| Slovenia | The #OstaniZdrav mobile application | Y |  |  | <https://www.gov.si/en/topics/coronavirus-disease-covid-19/the-ostanizdrav-mobile-application/> |
| Spain | Radar COVID | Y |  |  | <https://radarcovid.gob.es/> |
|  | SpTH |  |  | Travel Form | <https://play.google.com/store/apps/details?id=com.atos.spain.th&hl=en&gl=US> |
| Sweden | Resklar app | The deployment of a contact tracing app is not foreseen. |  | Travel Advice | <https://www.swedenabroad.se/sv/om-utlandet-f%C3%B6r-svenska-medborgare/nederl%C3%A4nderna/reseinformation/ud-resklar-appen/> |
| Switzerland | SwissCovid app | Y |  |  | <https://www.bag.admin.ch/bag/en/home/krankheiten/ausbrueche-epidemien-pandemien/aktuelle-ausbrueche-epidemien/novel-cov/swisscovid-app-und-contact-tracing.html> |
| EU | Re-open EU |  |  | Y - Travel advice | <https://apps.apple.com/us/app/re-open-eu/id1531322447> |
|  | EU Digital COVID Certificates |  | Y |  | [Create your Digital EU Covid Certificate Pass \| GetCovidPass.eu](https://www.getcovidpass.eu/) |

**Supplementary Figure 1.** Word cloud of the languages used for providing information on travel requirements during COVID-19. (A) Main languages (N=34 unique languages) and (B) additional languages available some on request (N=76). Analyzed using Voyant Tools. <https://voyant-tools.org/>


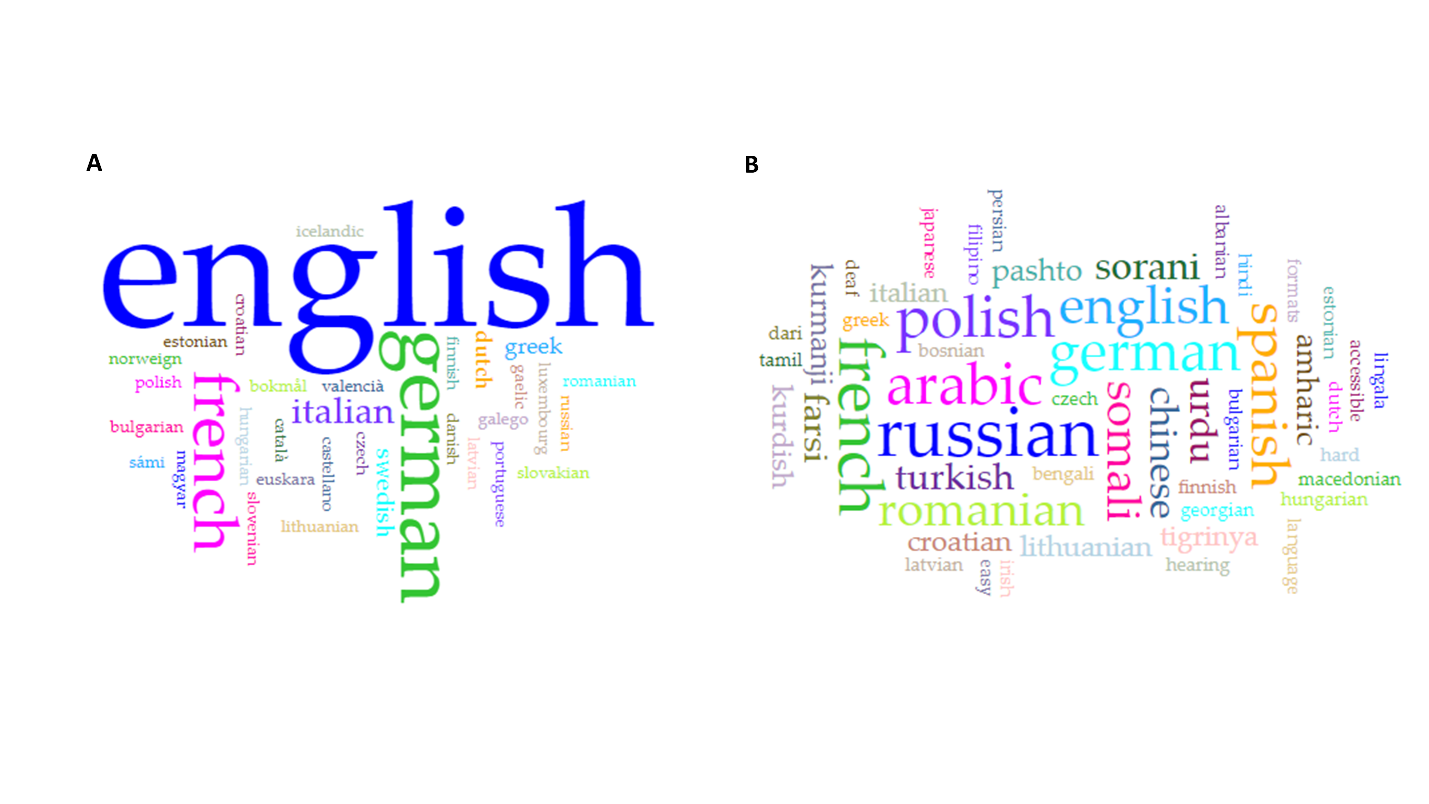


1. EuropeanCommission. Mobile contact tracing apps in EU Member States2021 Jul 31 2021. Available from: <https://ec.europa.eu/info/live-work-travel-eu/coronavirus-response/travel-during-coronavirus-pandemic/mobile-contact-tracing-apps-eu-member-states_en>.
